# Neural Mechanisms and Alterations of Sweet Sensing: Insights from Functional Magnetic Resonance Imaging Studies

**DOI:** 10.3390/life15071075

**Published:** 2025-07-05

**Authors:** Tobias Long, Colette C. Milbourn, Alison Smith, Kyaw Linn Su Khin, Amanda J. Page, Iskandar Idris, Qian Yang, Richard L. Young, Sally Eldeghaidy

**Affiliations:** 1 Division of Food, Nutrition and Dietetics, School of Biosciences, The University of Nottingham, Sutton Bonnington Campus, Loughborough LE12 5RD, UK; tobias.long@nottingham.ac.uk (T.L.);; 2 Sir Peter Mansfield Imaging Centre, School of Physics and Astronomy, The University of Nottingham, University Park, Nottingham NG7 2RD, UK; 3 Centre of Metabolism, Ageing & Physiology, Nottingham Biomedical Research Centre (BRC), School of Medicine, The University of Nottingham, Derby DE22 3DT, UK; 4 Lifelong Health, South Australian Health and Medical Research Institute (SAHMRI), Adelaide, SA 5000, Australia; 5 School of Biomedicine, The University of Adelaide, Adelaide, SA 5005, Australia; 6 Adelaide Medical School, The University of Adelaide, Adelaide, SA 5005, Australia; 7 Centre of Research Excellence in Translating Nutritional Science to Good Health, The University of Adelaide, Adelaide, SA 5005, Australia

**Keywords:** neuroimaging, fMRI, BOLD, sweet sensing, sweeteners, obesity, diabetes

## Abstract

Sweet sensing is a fundamental sensory experience that plays a critical role not only in food preference, reward and dietary behaviour but also in glucose metabolism. Sweet taste receptors (STRs), composed of a heterodimer of taste receptor type 1 member 2 (T1R2) and member 3 (T1R3), are now recognised as being widely distributed throughout the body, including the gastrointestinal tract. Preclinical studies suggest these receptors are central to nutrient and glucose sensing, detecting energy availability and triggering metabolic and behavioural responses to maintain energy balance. Both internal and external factors tightly regulate their signalling pathways, and dysfunction within these systems may contribute to the development of metabolic disorders such as obesity and type 2 diabetes (T2D). Functional magnetic resonance imaging (fMRI) has provided valuable insights into the neural mechanisms underlying sweet sensing by mapping brain responses to both lingual/oral and gastrointestinal sweet stimuli. This review highlights key findings from fMRI studies and explores how these neural responses are modulated by metabolic state and individual characteristics such as body mass index, habitual intake and metabolic health. By integrating current evidence, this review advances our understanding of the complex interplay between sweet sensing, brain responses, and health and identifies key gaps and directions for future research in nutritional neuroscience.

## 1. Introduction

All animals share an innate preference for sweet-tasting compounds, driven by an evolutionary need to seek energy sources [1,2]. This preference stems from a strong connection between food intake, gut hormone release, and the activation of brain regions associated with food intake and reward. Early research identified the hypothalamus as a key regulator of calorie and nutrient intake [3,4], with hormonal connections to appetite and satiety regulators, as well as neural connections to other brain areas, playing a crucial role in controlling homeostatic energy balance [5,6,7]. This complex gut–brain interaction has been essential for human survival throughout evolution. Our current understanding of the gut–brain interaction in the context of food intake is still in its early stages, and further investigation of the brain responses to nutrient sensing, ingestion and metabolism is essential. However, food intake is influenced not only by homeostatic mechanisms but also by sensory perception, reward processing, motivation, and emotional regulation [8,9]. In the modern environment, excessive consumption of sugar-sweetened foods and beverages is linked to weight gain and type 2 diabetes (T2D) [10,11], contributing to the global burden of metabolic diseases [12,13]. Understanding the neural mechanisms underlying sweet sensing is therefore crucial for informing future research and developing effective strategies to manage and prevent metabolic diseases.

Over the past three decades, advancements in functional neuroimaging techniques, particularly functional magnetic resonance imaging (fMRI), have provided valuable insight into central mechanisms underlying food-related perception, reward, and metabolism in the human brain. This review aims to explore the current research landscape focusing on sweet-related responses in the human brain, assessed through fMRI techniques. We begin with an overview of the sweet-sensing pathways and fMRI methods. Next, we highlight fMRI studies investigating oral and intestinal sweet sensing processing, including alterations to these pathways in obesity and T2D. Finally, we provide an outlook on the state of sweet sensing research and its implications for future approaches to managing metabolic health conditions.

## 2. Sweet Sensing Mechanisms

### 2.1. Sweet Taste Receptors

Taste, or gustatory sensation, is detected by taste receptor cells (TRCs), which are mainly located in taste buds throughout the oral cavity, including the tongue, palate, pharynx, and epiglottis. Taste buds are onion-like structures, each containing 50–100 taste cells, and are mostly found on the tongue’s papillae. These papillae come in three main types—fungiform papillae located on the anterior tongue, circumvallate papillae located medially on the rear tongue, and foliate papillae located laterally on the rear tongue. When food or drink is consumed, a small proportion of the food material dissolves in saliva, diffuses into the taste pore and interacts with the surface of the taste cells. Each of the five recognised taste qualities (sweet, salty, sour, bitter and umami) is identified by a specific receptor or ion channel on the surface of the TRC, each with a distinct transduction mechanism. Recent evidence suggests the existence of additional taste qualities, such as fat, starch, kokumi and metallic, though their underlying mechanisms and classification remain the subject of ongoing research and debate [14,15,16].

**Figure 1:**
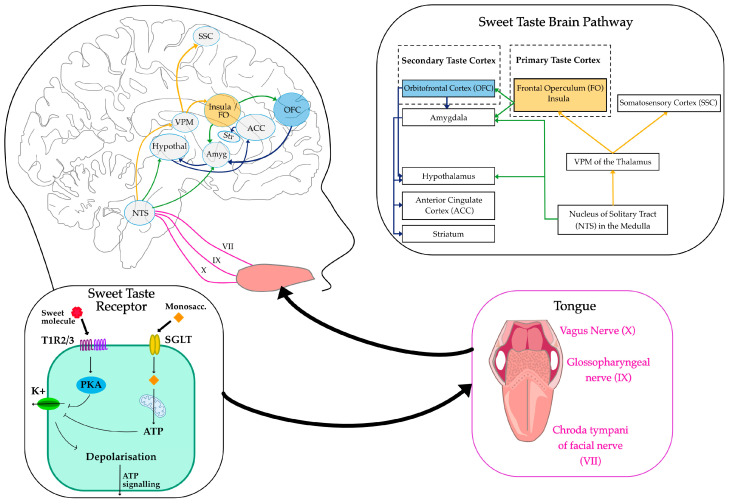
Neural Pathways of Sweet Taste Perception. The schematic illustrates the sweet taste transduction pathway from peripheral taste receptors to cortical brain regions involved in sweet taste processing. Sweet taste is detected by T1R2/3 or SGLT receptors on the tongue, leading to depolarisation of taste cells and further signalling via Protein Kinase A (PKA) and Adenosine Triphosphate (ATP) intermediary signals. Sensory signals are transmitted via the chorda tympani (VII), glossopharyngeal (IX), and vagus (X) nerves to the nucleus of the solitary tract (NTS) in the medulla. From there, projections reach the ventral posteromedial nucleus (VPM) of the thalamus and then the primary taste cortex, which includes the insula and frontal operculum (FO), somatosensory cortex (SSC). Secondary taste regions such as the orbitofrontal cortex (OFC), amygdala (Amyg), anterior cingulate cortex (ACC), striatum (Str), and hypothalamus (Hypothal) are also activated. Mitochondria object courtesy of BioRender, https://BioRender.com/60eau3n (accessed on 1 July 2025).

TRCs are classified into four morphologically and functionally distinct types (types I–IV). Evidence from molecular biology studies has shown that only type II cells are involved in sweet taste sensing. These cells express G-protein-coupled receptors (GPCRs) that detect sweet, bitter and umami tastants (see Figure 1). Specifically, a heterodimer of the taste receptor type 1 members 2 and 3 (T1R2/T1R3) has been identified as the sweet taste receptor (STR) following its characterisation in 2001 [17]. These STRs are stimulated by sweet-tasting compounds, including both nutritive and non-nutritive sweeteners (NNS). In addition to the STR-dependent pathway, there are STR-independent pathways that detect caloric sweeteners but not NNS. Two such pathways include the K_ATP_-dependent pathway and the sodium-glucose co-transporter (SGLT) family pathway. Both pathways have been implicated in sweet sensing and may serve distinct functions from the STR-dependent mechanism [18], suggesting a possible difference in neural activation and behavioural response between caloric and NNS. Signal transduction downstream of STR activation is out of the scope in this review; for a more detailed overview, see [19].

Interestingly, taste receptors are not only present in the oral cavity but are also expressed throughout the gastrointestinal tract, including in the stomach, intestines, and pancreas [20]. Members of the taste receptors from the T1R and T2R families are found throughout the body in organs such as the brain, kidney, liver, testes and thyroid [21]. The functions of many of these extraoral taste receptors are not completely understood. Gut-expressed taste receptors (such as T1R2/T1R3) are some of the most well-understood extraoral taste receptors. They are structurally like those on the tongue and respond to both nutrients and non-nutritive compounds [22]. Located primarily on intestinal enteroendocrine cells, these receptors play a central role in the regulation of key gut hormones such as the incretin hormones GLP-1 (glucagon-like peptide-1) and GIP (glucose-dependent insulinotropic peptide), which are crucial for appetite regulation, insulin secretion, and glucose metabolism [23]. This gut “taste system” operates as a chemosensory extension of the oral taste system, transmitting signals to the brain via the vagus nerve and endocrine pathways. In doing so, it plays a role in regulating satiety, energy homeostasis, and long-term food preferences [24].

### 2.2. Sweet Taste Pathways

Neurophysiological studies in animals have demonstrated that taste signals are transmitted via cranial nerves (VII, IX, and X) to a gustatory nucleus within the rostral nucleus of the solitary tract (NTS) in the medulla oblongata in the brainstem, the first central relay for taste information, and is also responsible for swallowing and salivatory reflexes. Neurons in the NTS integrate taste signals and project to multiple brain regions, including the ventroposterior medial nucleus (VPM) of the thalamus, which acts as the principal relay to the gustatory cortex [25]. The VPM also contains a topographic representation of the oral cavity. From the thalamus, taste information is transmitted to the primary gustatory cortex, located in the anterior insula and frontal operculum, where taste quality, intensity, and affective value are encoded [26].

Two prominent theories have been proposed to explain how taste information is encoded and transmitted to the primary gustatory cortex: the “labelled lines” and “combinatory coding” models. The labelled lines theory suggests that individual TRCs and their associated neural pathways are selectively tuned to specific basic taste qualities and transmit signals via dedicated, independent pathways to the brain [27]. In contrast, the combinatorial coding model suggests that taste qualities are encoded by patterns of activity across a population of broadly responsive TRCs and neurons, rather than being relayed through discrete channels [28]. Current evidence indicates that both models may contribute to taste processing at different levels of the neural hierarchy [27]. While labelled lines may be more prominent in peripheral taste pathways, combinatory coding likely plays a greater role in central processing [29].

Beyond the primary gustatory cortex, the orbitofrontal cortex (OFC) has also been shown in electrophysiology studies to act as a higher-order taste centre involved in encoding emotional values of the taste stimulus [30]. OFC activity is believed to be modulated by motivational state, responding to hunger and not satiety [31]. It has also been postulated that the lateral part of the OFC may play a role in hedonic judgements. Additionally, taste circuits interact with limbic structures such as the amygdala, anterior cingulate cortex (ACC), striatum, and hypothalamus, linking taste perception to reward processing, which involves the hedonic and motivational properties of taste stimuli [32], see Figure 1. These interconnected neural pathways ensure that taste perception is a complex experience that integrates hedonic, motivational, and homeostatic factors [33,34].

Recent graph theory analyses in anaesthetised, non-human primates [35] have further elucidated the structure of gustatory networks, proposing a three-part connectome comprising medial, lateral and subcortical modules. Despite this model supporting the human fMRI findings on the sweet-sensing pathways (for example, exhibiting insula-OFC connectivity), its applicability to conscious human responses requires validation. Future research could investigate such modularity in human fMRI studies, particularly in the context of metabolic disorders and their effects on the proposed connectome.

### 2.3. Sweet-Tasting Molecules

Sweet stimuli are many and varied. The most familiar are the monosaccharide hexoses glucose, fructose and their disaccharide sucrose (table sugar). These simple carbohydrates are rapidly absorbed into the blood and used for energy. Another class of sweet stimuli are non-caloric or NNS, a group of natural and synthetic molecules that activate the sweet taste pathways without containing monosaccharides as an energy source (although some NNS, such as aspartame and steviol glycosides, are broken down) [36]. Typical examples of these molecules include those abundant in the human food supply, such as sucralose (known as E955), aspartame (E951), and erythritol (E968). NNS are a very diverse group of small molecules and proteins that are sweeter than sugars [37,38]. These non-nutritive sources of sweetness are widely formulated in diet-related products and drinks, aimed at reducing the consumption of caloric sugars in an obesogenic environment [39]. However, their effects on long-term health are not yet fully understood, and there is ongoing debate and research over their impact on human health [40,41]. In 2023, the World Health Organisation (WHO) advised against the use of NNS for weight control, citing insufficient long-term benefits and potential adverse effects [42]. Another class of sweet-tasting molecules subject to ongoing research are rare sugars. These are monosaccharides and disaccharides that are rarely found in nature. A full understanding of their biological functions is lacking, but preliminary evidence in human trials suggests they have beneficial effects on glycaemic control and weight loss [43]. However, the neural mechanisms are relatively unexplored, and to date, no fMRI studies have assessed the impact of rare sugars on sweet taste pathways in the human brain.

## 3. fMRI of Sweet Sensing in the Human Brain

### Principles of fMRI

**Figure 2:**
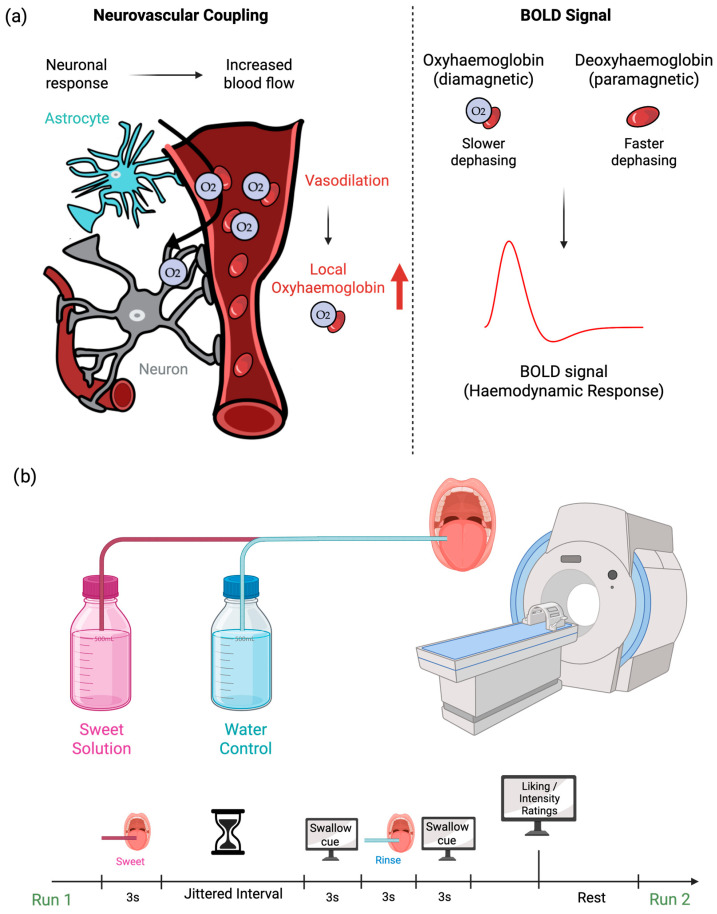
A schematic of fMRI paradigm assessing brain responses to sweet taste stimuli. (**a**) Neuronal activation from sweet taste stimulus increases metabolic demand, causing vasodilation and a rise in local oxygenated haemoglobin, measured as a BOLD signal. (**b**) Example of sweet taste fMRI protocol, sweet and water solutions are delivered during fMRI scanning in multiple runs. Each run includes a sweet stimulus, water rinse, swallowing cue, and subjective ratings of taste intensity and liking. Created in BioRender, https://BioRender.com/m6c6dcf (accessed on 1 July 2025).

Blood oxygen level-dependent functional MRI (BOLD-fMRI) is currently the most widely used functional neuroimaging technique for mapping changes in brain activity at both cortical and subcortical levels, including studying food perception and metabolism. A key advantage of BOLD-fMRI is that it is a non-invasive technique, allowing serial or repeated scans to be collected from the same individual, without the need for radiation. The BOLD signal reflects changes in blood oxygenation, as oxygenated and deoxygenated blood have different magnetic properties. This provides an indirect measure of neuronal activity where an increase in signal indicates increased neuronal activity, and a decrease in signal indicates decreased neuronal activity. When food-related cues activate gustatory neurons, these signals propagate to the brain, and this neuronal activity subsequently triggers an increased demand for oxygen and glucose. This initiates a cascade of physiological responses, collectively referred to as neurovascular coupling, including vasodilation and changes in cerebral blood flow (CBF), cerebral blood volume, and the cerebral metabolic rate of oxygen consumption [44]. These changes increase the local ratio of oxyhaemoglobin to deoxyhaemoglobin in the activated brain regions, which can be detected and visualised by fMRI, as illustrated in Figure 2.

Most fMRI studies investigate task-induced changes in the BOLD signal (brain activation) to localise and characterise brain regions involved in sweet sensing and metabolism. Its high spatial resolution enables precise mapping of sweet taste pathways and their integration with reward-related networks in the human brain. A common approach is the use of ‘block design’, in which periods of stimulus presentation or task performance alternate with rest blocks (no task/rest) or a control condition. In a typical taste fMRI study, taste stimuli are delivered into the mouth through plastic tubing for a fixed duration, followed by a rest period. The stimuli are typically held in the mouth for approximately 3 s, followed by a rest period of about 10 s; however, the duration of the stimulus may vary between studies. Signal averaging and statistical analysis are then used to identify activation patterns associated with the stimulus. Brain responses to food or food-related cues can be measured under different physiological states, such as the fasted state (baseline) vs. the fed state (post-ingestion/post-infusion). In addition to task-based approaches, functional connectivity is used to assess the statistical relationship or synchronisation between neural activities across different brain regions, either during task performance or at rest. Resting-state fMRI (rs-fMRI) is used for the study of the brain’s intrinsic functional architecture without the need for an explicit task [45,46]. It is commonly employed to explore connectivity differences under varying metabolic states, such as between fasted and fed conditions.

In addition to the BOLD signal, arterial spin labelling (ASL) is another fMRI technique used to quantify CBF directly. In ASL, hydrogen in the water of arterial blood is magnetically labelled before reaching the tissue of interest. After a short post-label delay, the labelled blood (acting as an endogenous tracer) flows into the region of interest and exchanges with brain tissue water. At this point, an image is acquired (the label image). A corresponding control image, acquired without labelling the blood, is then subtracted from the label image to produce a perfusion-weighted image, which can be quantified in terms of CBF (in mL/100 g/min). Like rs-fMRI, ASL data, or the quantification of CBF, are typically acquired before and after the infusion or ingestion of a test meal [47]. Brain responses are then compared across meals, time points, or study groups.

## 4. fMRI of Oral Sweet Sensing

**Figure 3:**
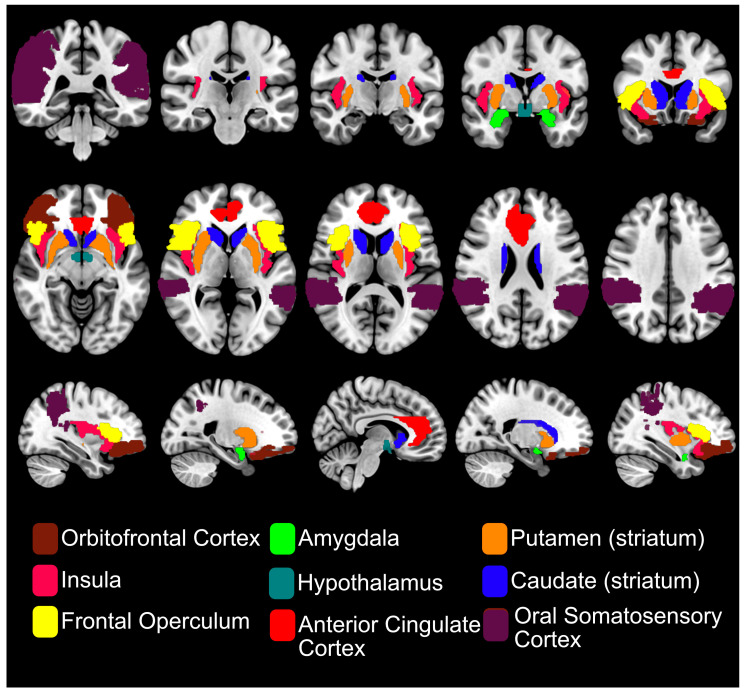
Brain areas frequently reported in sweet-taste fMRI studies reflect the integration of sensory (insula, frontal operculum, oral somatosensory cortex), hedonic (striatum, amygdala, anterior cingulate cortex, orbitofrontal cortex, and homeostatic (hypothalamus) components of sweet taste perception.

Taste fMRI is considered the gold standard for identifying the neural basis of taste perception and for mapping the topographical organisation of gustatory processing in the human brain [48]. Recent Activation Likelihood Estimation (ALE) meta-analyses of fMRI studies on sweet taste found consistent activation in gustatory, somatosensory, and reward-related brain areas [49,50,51], reflecting the sensory, hedonic, and homeostatic components of taste processing (see Figure 3). In an ALE meta-analysis by Roberts et al. [49], consistent BOLD responses to both sugars and NNS were reported in the insula and frontal operculum across all 15 included fMRI studies. Additional regions, such as the amygdala, were also reported. In one of the first fMRI studies to examine the cortical response to pleasant (glucose) and aversive (salt) taste stimuli, the amygdala showed preferential activation to sweet taste, highlighting its role in reward processing [52]. Other frequently reported regions in taste fMRI studies include the ACC, striatum, thalamus, hypothalamus, precentral gyrus and both primary and secondary somatosensory cortices [53]. See Figure 3 for brain areas frequently reported in fMRI studies.

### 4.1. fMRI of Individual Variations in Sweet Perception

Individual variation in sweet taste perception has also been explored using fMRI. For example, Rudenga et al. investigated brain responses to sweet stimuli in ‘sweet likers’ and ‘sweet dislikers’ [54]. These ‘sweet liking status’ classifications are based on participants’ liking of varying concentrations of sweet solutions [55]. Both groups showed activation in taste and reward-related brain regions, as well as the ventromedial prefrontal cortex (vmPFC) and thalamus. However, sweet likers demonstrated greater vmPFC BOLD responses than sweet dislikers, suggesting that the vmPFC plays a role in encoding sweet preference, rather than sweet intensity or quality. The dose-independent response of the amygdala, previously observed in other studies [56], was also reported in this work. However, in contrast to earlier findings [57,58], no dose-dependent responses were observed in the taste or other reward regions. These discrepancies may be due to differences in study populations, solution concentrations, tasting protocols, or the specific types of taste stimuli used. Further research is needed to better understand how sweet intensity and liking modulate brain responses across diverse populations.

In addition to hedonic preference, other phenotypic variations have also been linked to differences in sweet perception. For instance, studies examining the thermal taster phenotype, a condition where individuals perceive taste when the tongue is thermally stimulated, have shown that thermal tasters demonstrate increased brain responses to both cold and ambient sweet stimuli in regions associated with taste and oral somatosensory processing [59]. In addition, some studies have reported associations between sweet liking classification and PROP (6-n-propylthiouracil, a bitter compound) taste status; however, findings are mixed [60,61], and the understanding of underlying neural mechanisms remains limited. Exploring the neural bases of interactions between different taste phenotypes using fMRI is needed. Moreover, accounting for individual variation in taste fMRI studies is critical, as such variability can influence statistical power.

### 4.2. Brain Activation of Caloric Sweeteners vs. NNS

A key question in fMRI studies is how the brain distinguishes between caloric sweeteners and NNS that are matched in sweetness. Frank et al. demonstrated that while participants were unable to distinguish between matched sucrose and sucralose solutions, brain activation varied by type of sweetener [62]. Both sweeteners activated the primary taste pathway, but only sucrose elicited activation in dopaminergic midbrain regions, which are associated with pleasurable reward processing. Compared to sucralose, sucrose also evoked stronger responses in the anterior insula, frontal operculum, striatum, ACC, and prefrontal cortex. The authors proposed that this broader activation in food reward-related regions may reflect sucrose’s ability to engage additional mechanisms beyond T1R3 receptor activation, in contrast to sucralose. Further support for the brain’s ability to differentiate between caloric sweeteners and NNS comes from Smeets et al. [63], who showed that the amygdala showed decreased activation to caloric (sucrose) versus NNS (aspartame, acesulfame K) sweet drinks. Moreover, only the caloric versions modulated taste-related activation in the striatum, reinforcing the link between energy content and reward processing.

Chambers et al. extended these findings by showing that the human brain can distinguish between caloric sweeteners (glucose and maltodextrin) and NNS (saccharin) independent of perceived sweetness, by utilising taste-matched solutions [64]. While all sweeteners activated the frontal operculum and prefrontal cortex, only glucose led to additional activation in reward-related areas like the ACC and striatum. Interestingly, maltodextrin, despite being less sweet than glucose, produced a neural activation pattern similar to glucose, suggesting that the brain can detect caloric content independently of sweetness. These results challenge the assumption that sweet taste alone drives central reward and support the concept of separate neural coding for caloric sweeteners versus NNS.

The neural effects of caloric sweeteners and NNS have also been examined in the context of more complex nutrient meals/drinks. For example, Van Opstal et al. [65] compared sweetened nutrient shakes containing fat and protein. They found that glucose and fructose-sweetened shakes significantly reduced the BOLD signal in several brain areas, whereas allulose and sucralose-sweetened shakes did not. Fructose activated more frontal brain regions than glucose, and both sugars reduced activity in the insula. Furthermore, only glucose increased activity in the salience network, which is associated with feeding behaviour and reward. These findings indicate that satiety and reward-related responses may differ between caloric sweeteners and NNS even when embedded in mixed-nutrient meals. The fact that both sucralose (NNS) and allulose (low-energy carbohydrate) failed to elicit significant brain responses in reward areas supports the idea that the brain’s reward response is closely linked to the energy content of the sweetener [63].

More recently, Budzinska et al. investigated the neural and subjective responses to taste-matched solutions of sucrose, sucralose, and erythritol in healthy male participants [66]. Although all sweeteners were matched for perceived sweetness, sucrose and sucralose were rated as more pleasurable than erythritol. While univariate analyses did not reveal significant differences in activation across traditional taste and reward regions (e.g., insula, OFC, striatum), multivariate pattern analysis uncovered distinct neural signatures for each sweetener that predicted individual liking. This suggests that even when sweetness intensity is controlled, the brain processes sweeteners differently, likely reflecting differences in expected or experienced post-ingestive reward. Interestingly, unlike many other NNSs, erythritol has been shown to significantly increase GLP-1 and cholecystokinin levels (satiety hormones) and decrease plasma ghrelin levels (hunger hormone) [67,68], mimicking the effects of sucrose and glucose. This suggests that the brain integrates both sensory and physiological signals when evaluating sweet stimuli. This further emphasises the complexity of sweetener processing in the human brain and highlights the importance of considering both caloric content and post-ingestive feedback in understanding sweet taste perception.

### 4.3. Alterations in Brain Responses to Sweet Taste Following Habitual NNS Consumption

The lack of energy in NNS juxtaposes the evolutionary association between caloric intake and sweet taste. This mismatch may influence brain reward circuitry over time. Emerging evidence has raised concerns about potential alterations in brain responses to sweet taste resulting from habitual NNS consumption [38,39,40,41]. For example, the effect of habitual consumption of diet soft drinks on reward-related brain activity in response to sucrose and saccharin was compared in two groups: habitual diet soda drinkers and non-drinkers [69]. Interestingly, habitual diet soda drinkers showed greater activation in reward-related brain regions, including the VTA, midbrain, caudate, and amygdala, in response to both caloric and non-caloric sweet stimuli. This contrasts with earlier findings in non-habitual NNS users, where NNSs typically elicited lower or no activation in these same regions compared to caloric sweeteners [61,63]. One possible explanation is that habitual NNS consumers may have adapted to derive similar hedonic value from NNSs as from sugar, resulting in heightened brain responses to these sweeteners. As a result, repeated exposure could condition the brain to respond more strongly to NNSs.

To test whether such adaptations could emerge after repeated short-term exposure, Griffioen-Roose et al. assessed the impact of a four-week conditioning period on reward-related brain responses to nutrient-rich (yoghurt-based) or nutrient-empty (soft drink), sweetened with either sugar or NNS [70]. Although participants reported greater satiety and reduced hunger after the yoghurt drinks compared to soft drinks, no significant changes in brain activity were observed for either sweetener type. These results suggest that short-term NNS exposure may have minimal effects on the brain’s reward system, supporting the use of NNSs in sugar-reduction strategies. However, the four-week conditioning period is brief compared to long-term habitual use in real-life contexts, and findings should be interpreted with caution.

In contrast, Dalenberg et al. reported differences in their study participants who consumed beverages sweetened with sucrose, sucralose, or sucralose combined with maltodextrin for two weeks [71]. Only the group consuming the sucralose + maltodextrin beverage showed significant effects, with a reduction in insulin sensitivity accompanied by decreased activity in the insula, ACC, VTA, putamen, and areas within the superior temporal gyrus and postcentral gyrus. These alterations were specific to sweet taste responses, as responses to other basic tastes remained unchanged. Furthermore, neither sucralose nor maltodextrin alone produced any significant effects.

These findings challenge previous support for the “sweet uncoupling hypothesis,” which suggests that adverse effects arise from decoupling sweet taste from caloric content [72,73]. Instead, these results suggest that the brain may not respond to NNSs alone, but to the combination of NNS and caloric intake in context. Hence, NNS may alter glucose metabolism and modulate neural reward pathways, resulting in altered food craving. It is important to note that this study was conducted in healthy individuals whose insulin sensitivity was temporarily impaired by the combination of NNS and calories, not due to chronic insulin resistance; responses might be different for chronic metabolic conditions such as obesity or diabetes. Taken together, these findings illustrate the complexity of NNS-brain interactions and highlight the need for further long-term studies. Factors such as habitual consumption, nutritional context, and individual metabolic state likely play key roles in shaping the brain’s response to sweeteners and should be considered in future research.

## 5. fMRI of Post-Oral Ingestive Sweet Responses

In addition to task-based fMRI, post-ingestive responses to sweet stimuli can be assessed using resting-state functional connectivity (rsFC) or CBF measurements, collected before and after the consumption of sweet drinks/test meals. These methods provide insights into how nutrient ingestion modulates brain networks over time, offering an understanding of the metabolic and satiety-related effects of different sweeteners. Although multiple studies measuring rsFC or CBF have assessed the response to caloric sweeteners, studies comparing these to NNS are lacking and offer research potential.

Given that metabolic state (fasted vs. fed) influences hunger and food motivation, it is expected to affect neural processing of sweet stimuli. Wright et al. explored the rsFC of the insula and hypothalamus following glucose ingestion under fasted and fed states [74]. In the fasted state, ingesting glucose increased rsFC between the insula and superior frontal gyrus, as well as between the hypothalamus and inferior frontal gyrus. In the fed state, ingesting glucose increased rsFC between the insula and posterior parietal cortex, and between the hypothalamus and superior parietal cortex and cingulate cortex. These results highlight the dynamic modulation of food-related brain regions involved in metabolic state, with changes in rsFC correlating with blood glucose levels. Al-Zubaidi et al. also demonstrated significant metabolic state-dependent changes in brain connectivity, assessed by local and global connectivity and fractional amplitude of low-frequency fluctuations (fALFF) [75]. The fasted state was associated with significant differences in fALFF in the posterior cingulate cortex, thalamus, and anterior precuneus. A follow-up study [76] included plasma glucose and insulin as covariates. Results showed that changes in plasma insulin, but not glucose, were associated with increased rsFC between the insula and superior frontal gyrus, suggesting a role for insulin in signalling energy deficiency and driving food-seeking behaviour. These findings differ slightly from Wright et al., who found glucose-related rsFC changes in the insula but did not include insulin as a covariate [74]. To address such discrepancies, future studies may benefit from using glucose or insulin clamp techniques to more precisely control metabolic variables during scanning.

Resting-state methods have also been used to assess differential brain responses to various sweeteners. For example, ingestion of glucose and fructose has been compared using rs-FC [77]. Glucose consumption increased rsFC between the caudate and putamen (within the striatum) and between the precuneus and lingual gyrus; additionally, these changes correlated with plasma insulin levels. In contrast, fructose consumption increased rsFC within the basal ganglia and limbic network. These neural differences were reflected in subjective appetite ratings: glucose increased feelings of fullness and reduced the desire to eat, whereas fructose had fewer effects.

A complementary task-based study by Luo et al. [78] assessed food cue reactivity after glucose or fructose ingestion. Fructose ingestion led to greater activation in the visual cortex, possibly reflecting increased attention to high-calorie foods. Higher activation was also seen in the OFC and striatum during food cue presentation following fructose ingestion compared to glucose. However, the striatal effect was only marginally significant. These results support the idea that fructose is less satiating than glucose, potentially leading to greater total energy intake.

Another fMRI approach has examined the BOLD response following glucose ingestion via peroral tubing, allowing test drinks to be delivered directly to the stomach. In healthy individuals, the glucose infusion inhibited BOLD activation in the lower posterior hypothalamus (LPH), corresponding to the ventromedial nucleus, but showed no significant effects in the upper anterior hypothalamus (UAH), which corresponds to the paraventricular nucleus [79]. This inhibition in activation was dose-dependent, as higher concentrations of glucose were associated with a greater inhibition of the hypothalamic signal. Liu et al. [80] also reported that BOLD signal decreased in regions associated with feeding and reward, including the gustatory cortex, OFC, ACC, hippocampus, thalamus and occipital cortex. These changes in BOLD signal correlated with plasma insulin levels. A follow-up study showed further decreases in BOLD signal in the brainstem and cerebellum, though hypothalamic changes were not reported [81].

To further disentangle the effects of sweetness and caloric content on post-ingestive brain responses, Smeets et al. [63] compared the brain’s response to four drinks given orally: sweet-caloric (glucose), NNS (aspartame), non-sweet-nutritive (maltodextrin), and a non-sweet, non-nutritive water control. This was the first study to separate the effects of sweetness and caloric content systematically. A decrease in hypothalamic BOLD signal was observed only for glucose, not for maltodextrin or aspartame. This finding challenges the earlier hypothesis that hypothalamic inhibition was driven solely by circulating insulin levels [82], since maltodextrin, a carbohydrate that elicits insulin secretion, did not inhibit hypothalamic activity. These results indicate that both sweetness and caloric content must be present to modulate hypothalamic responses, highlighting the importance of integrated sensory and post-ingestive signals in regulating food intake. Importantly, aspartame is rapidly hydrolysed after ingestion [83], and therefore its effectiveness for measuring post-ingestive responses in this study is questionable.

## 6. fMRI Studies of Gastrointestinal Sweet Sensing

Emerging research highlights the important role of the gut–brain axis in modulating central reward pathways, even in the absence of conscious taste perception. This research shows that the regulation of sweet and glucose metabolism via the gut–brain axis is partly mediated by intestinal STRs. By isolating the activation of intestinal STRs from oral taste responses, fMRI studies can help identify brain regions involved in post-oral sweet sensing of glucose and other sweeteners. However, it is important to consider the effects of gastric stretch receptors on satiety and appetite signalling to the brain, in addition to the effects of intestinal STRs. To control for these effects, the inclusion of a control solution or infusion should be used [84]. It is essential to distinguish intragastric (IG) infusion from intravenous (IV) infusion methods, such as those used in [85], as IV glucose bypasses intestinal STRs entirely. Nevertheless, IV studies can serve as useful comparators for distinguishing between responses to energy content versus intestinal sweet sensing, but these findings are outside the scope of this review.

Like previous observations with oral glucose ingestion, IG glucose infusion led to a reduction in BOLD signal in the hypothalamus and midbrain compared to a saline control [86]. In contrast, IG fructose produced a smaller reduction in these same regions relative to water, whilst other homeostatic and hedonic brain areas also showed decreased activity [87]. This supports previous evidence that fructose does not cause the same satiating effects as glucose after ingestion. Despite these neural differences, no significant changes in subjective hunger, food cravings, or emotional responses were reported [88,89], raising questions about the translation of these brain activity patterns to real-world behavioural outcomes. It is important to note that the dose of fructose used (25 g) was lower than the standard 75 g used in IG glucose protocols and previous oral studies, due to fructose absorption limits. This dosage difference may partially explain the weaker neural and behavioural effects observed with fructose, which warrants further investigation.

The brain responses to IG delivery of a complex nutrient emulsion in the form of chocolate milk vs. water were also explored [89]. Both water and chocolate milk were delivered via IG infusion and oral administration. IG infusion of either solution activated the midbrain, amygdala, hippocampus, and hypothalamus, suggesting that these regions respond to nonspecific IG stimuli. However, oral delivery of chocolate milk elicited stronger activation in the amygdala, precuneus, thalamus, and putamen compared to IG delivery. In contrast, IG delivery resulted in greater activation of the hippocampus and ACC. These findings indicate that reward and intake-related brain areas are more strongly engaged during oral intake than via IG infusion alone, emphasising the critical role of oral sensory stimulation in driving satiation and reward-related neural processing. Together, these studies highlight the importance of integrating intestinal sweet sensing into models of food intake and reward and highlight the distinct contributions of oral and post-oral phases in shaping eating behaviour and metabolic regulation.

## 7. Alterations in Sweet Sensing Responses in Metabolic Disorders

A key question considering the current epidemic of metabolic diseases is how sweet taste perception and associated brain responses differ from those in healthy individuals. Investigating these differences, using fMRI, provides valuable insight into how reward processing, appetite control, and nutrient sensing may be altered in disease states. Such findings are essential for advancing our understanding of the neurobiological mechanisms that contribute to dysregulated eating behaviour and impaired metabolic function.

### 7.1. fMRI of Alteration in Oral Sweet Sensing

Sensory and behavioural studies have reported altered taste perception in individuals with obesity [90] and diabetes [91], and fMRI studies have been conducted to understand the underlying neural mechanisms. Elevated BMI has been associated with an increased activation in the insula cortex and postcentral gyrus in response to sweet stimuli; however, activations in the striatum decreased as BMI increased [49]. This pattern suggests that individuals with a higher BMI, who are also at increased risk of impaired glucose metabolism, may exhibit heightened neural sensitivity to sweet stimuli in some regions, while also experiencing a reduced reward response in the striatum. Such a reduction in striatal activation may reflect a diminished reward signal, potentially prompting individuals to seek more intensely sweet foods to achieve the same hedonic effect, which contributes to overeating. In a recent fMRI study, the BOLD response to a sweet milkshake was assessed in the caudate and putamen across three groups: metabolically healthy lean (MHL), metabolically healthy obese (MHO), and metabolically unhealthy obese (MUO) individuals [92]. The MHL group showed a positive BOLD response, the MUO group showed a primarily negative BOLD response, and the MHO group demonstrated an intermediate response. Interestingly, participants in the MUO group underwent a second fMRI scan following a weight loss intervention. After weight loss, taste-induced activation in the caudate and putamen increased. These findings support the hypothesis that insulin resistance and obesity contribute to abnormal taste-related responses in the striatum, which may be partially reversible through weight loss.

### 7.2. fMRI of Alteration in Post-Oral Ingestive Glucose/Sweet Sensing

fMRI studies examining alterations in post-ingestive glucose or sweet sensing in metabolic diseases are limited. Most of these studies have focused on comparing individuals with obesity or elevated BMI to healthy controls, with only a single study to date, to our knowledge, has assessed the neuronal responses to post-ingestive glucose or sweet sensing in individuals with T2D [93]. This represents a significant gap in the literature and highlights the need for more comprehensive neuroimaging research to better understand how post-ingestive glucose/sweet sensing and its neural correlates are affected in metabolic disorders. Matsuda et al. reported altered hypothalamic function to glucose ingestion in lean versus obese individuals, both without T2D, using BOLD fMRI following the ingestion of a 75 g glucose solution [82]. Both lean and obese participants showed a reduction in BOLD signal in the hypothalamus (LPH and UAH) within 4–10 min of glucose ingestion, followed by a return to baseline. However, in individuals with obesity, the response in the LPH was significantly delayed compared to the lean group. This delayed and attenuated inhibitory response in the LPH may reflect a reduced satiation signal, leading to increased energy intake and contributing to weight gain [94]. In another study using a similar fMRI protocol, moderately obese individuals with T2D demonstrated a distinctly different hypothalamic BOLD response. In healthy participants, oral glucose ingestion led to the expected decrease in BOLD signal in both upper and lower hypothalamic regions, consistent with previous findings [79,80], However, this response was absent in individuals with T2D [93]. The absence of this BOLD signal decrease in the group with T2D suggests dysregulation of the hypothalamus’ function in processing glucose-related signals. This finding further supports the disruption in the central glucose signalling in metabolic diseases.

Chakravartti et al. extended these findings by examining how ingestion of sucralose versus sucrose or water affects hypothalamic CBF and rsFC across body weight groups [95]. In a large, diverse sample, they found that sucralose increased hypothalamic CBF relative to both sucrose and water, particularly in individuals with obesity and in women. Unlike sucrose, sucralose failed to reduce hypothalamic activity or hunger ratings, and instead increased connectivity between the hypothalamus and regions involved in motivation and somatosensory processing. These results reinforce the idea that the hypothalamus, which is critical for appetite and energy balance, responds not just to sweetness but also to the presence or absence of calories. The exaggerated hypothalamic response to sucralose in the absence of metabolic feedback may represent a form of neural mismatch that could contribute to altered appetite regulation with chronic NNS use.

### 7.3. fMRI of Alteration in Intestinal Sweet Sensing

A recent study by Simon et al. investigated hypothalamic and reward network reactivity to glucose metabolism in individuals with obesity using IG glucose infusion [96]. They supported the findings from Matsuda et al. [82] of delayed deactivation in the hypothalamus in individuals with obesity compared with healthy participants. In addition, they demonstrated decreased reactivity in the ventral striatum and amygdala in obesity compared to healthy weight participants.

## 8. Concluding Remarks and Future Perspectives

Recent advancements in fMRI have enabled the identification of the complex network of brain regions involved in sweet sensing and glucose metabolism. Improvements in spatial and temporal resolution, particularly through the use of multiband echo-planar imaging (EPI), which enables faster data acquisition with broader brain coverage, and multi-echo EPI, which enhances BOLD sensitivity by improving the contrast-to-noise ratio, have significantly advanced the quality of fMRI data [97], especially in regions susceptible to signal dropout and distortion, such as the OFC [98]. The development of ultra-high-field MRI (>3 T) has further enabled more precise anatomical segmentation and functional mapping of small subcortical structures involved in taste and metabolic regulation [99]. Additionally, ASL techniques provide quantitative measures of CBF, offering a quantitative measurement of neurovascular coupling during sweet taste processing. Resting-state fMRI studies contribute valuable information on baseline brain network organisation and its modulation by metabolic factors. These studies suggest that functional connectivity within gustatory and reward-related networks may be altered by body mass index, habitual sugar intake, and metabolic health, providing a complementary perspective to sweet taste fMRI investigations [100,101]. Research integrating task-based, resting-state, and ASL approaches deepens our understanding of the complex neural substrates of sweet sensing and their dysregulation in metabolic diseases.

While emerging evidence suggests that nutrient sensing in the gut, particularly the proximal small intestine, can influence central reward, satiety, and metabolic networks, neuroimaging studies directly examining how gut-derived signals modulate brain responses to sweet stimuli remain limited. It also remains unclear to what extent the brain’s response to intestinal glucose sensing differs with different metabolic states like obesity and T2D compared to healthy controls, or how such differences contribute to impaired glucose homeostasis, altered appetite regulation, or food preference. Additionally, molecules involved in gut–brain communication, such as serotonin (5-HT) and gamma-aminobutyric acid (GABA), play a key role in understanding the neurobiological mechanisms underlying responses to food and sweet intake [102,103]. Elucidating these mechanisms is crucial for developing targeted pharmacological or dietary interventions that leverage gut-sensing pathways to restore metabolic balance.

In addition, insights gained from fMRI can inform the development of targeted dietary and pharmacological strategies aimed at restoring healthy neural responses to sweet stimuli. For example, dietary interventions, such as modifying sweetener exposure or timing carbohydrate intake, can be evaluated for their ability to restore normal brain responses to sweet stimuli, particularly in individuals at risk for metabolic disease. fMRI can also be used to measure the efficacy of these interventions by tracking changes in activation and connectivity in sweet- and reward-related brain regions. Similarly, pharmacological agents aimed at modulating central or peripheral pathways, such as GLP-1 receptor agonists, GABA or serotonin modulators, or hypothalamic-targeting peptides, can be tested for their impact on brain responses to sweet stimuli, helping to optimise treatment for obesity and T2D. By linking metabolic signals with central neural outcomes, fMRI bridges the gap between nutritional or drug-based interventions and their underlying neurobiological mechanisms, supporting a more personalised and mechanistically informed approach to treating diet-related disorders.

In parallel, there have been increasing concerns in recent years over the widespread use of NNS, regarding their potential effects on glucose metabolism, gut microbiota, and appetite regulation. This has prompted the current WHO advice to avoid NNS use for weight loss or control of T2D [42]. A promising alternative is rare sugars. Rare sugars, such as allulose (D-psicose) and tagatose, are found naturally in small quantities in certain fruits and vegetables but can now be produced at scale through enzymatic processes [104]. These sugars closely mimic the taste and texture of traditional sucrose but provide fewer calories and have minimal impact on blood glucose and insulin levels [105,106]. Preliminary studies also suggest potential benefits for lipid metabolism, gut microbiota, and appetite control [107,108]. However, clinical data on human appetite and food intake are limited, and further research is needed to confirm these findings. Investigating brain responses to rare sugars using neuroimaging would be highly beneficial in understanding their effects on human appetite and potential use as alternatives to NNS.

To conclude, although significant progress has been made, our understanding of the biological and neurological mechanisms underlying sweet sensing remains incomplete. Existing research highlights strong connections between sweet taste responses and both homeostatic and hedonic brain areas. However, given the growing epidemic of T2D and obesity, there is a crucial need for more targeted research into how sweet taste, appetite regulation, and reward processing interact in these individuals.

## Data Availability

Not applicable.

## Abbreviations

The following abbreviations are used in this manuscript:

ACC	anterior cingulate cortex
ATP	adenosine triphosphate
BMI	body mass index
BOLD	blood oxygen level-dependent
CBF	cerebral blood flow
fALFF	fractional amplitude of low-frequency fluctuation
fMRI	functional magnetic resonance imaging
GLP-1	glucagon-like peptide-1
GPCR	G-protein-coupled receptor
IG	intragastric
IV	intravenous
LPH	lower posterior hypothalamus
MHL	metabolically healthy lean
MHO	metabolically healthy obesity
MUO	metabolically unhealthy obesity
NNS	non-nutritive sweetener
NTS	nucleus of the solitary tract
OGTT	oral glucose tolerance test
OFC	orbitofrontal cortex
PKA	protein kinase A
PNS	parasympathetic nervous system
ROI	region of interest
rsFC	resting-state functional connectivity
SGLT	sodium-glucose co-transporter
SNS	sympathetic nervous system
STR	sweet taste receptor
T1R2	taste 1 receptor member 2
T1R3	taste 1 receptor member 3
T2D	type 2 diabetes
TRC	taste receptor cell
UAH	upper anterior hypothalamus
vmPFC	ventromedial prefrontal cortex
VPM	ventroposterior medial nucleus
